# Association of metformin intake with bladder cancer risk and oncologic outcomes in type 2 diabetes mellitus patients: A systematic review and meta-analysis

**DOI:** 10.1097/MD.0000000000011596

**Published:** 2018-07-27

**Authors:** Jiao Hu, Jin-bo Chen, Yu Cui, Ye-wen Zhu, Wen-biao Ren, Xu Zhou, Long-fei Liu, He-qun Chen, Xiong-bing Zu

**Affiliations:** a Department of Urology; b Reproductive Medicine Center, Xiangya Hospital, Central South University, Changsha, China.

**Keywords:** bladder cancer, meta-analysis, metformin

## Abstract

**Background:**

Recent clinical trials indicated that metformin intake might play a protective role in the incidence and oncologic outcomes of various cancers. However, its protective effect on bladder cancer remains uncertain.

**Methods:**

We performed a meta-analysis to investigate the association between metformin intake and bladder cancer risk as well as oncologic outcomes in diabetes mellitus (DM) patients. A comprehensive literature search was performed using PubMed, Embase, and the Cochrane Central Search Library in December 2017. Hazard ratio (HR) with 95% confidence interval (CI) was pooled.

**Results:**

A total of 9 retrospective cohort studies with 1,270,179 patients were included. A meta-analysis revealed that metformin intake was associated with an increased recurrence-free survival (HR = 0.55, 95% confidence interval [CI] = 0.35–0.88; *P* = .01; *I*^2^ = 64%), improved progression-free survival (HR = 0.70, 95% CI = 0.51–0.96; *P* = .03; *I*^2^ = 33%), and prolonged cancer-specific survival (HR = 0.57, 95% CI = 0.40–0.81; *P* = .002; *I*^2^ = 0%). However, results demonstrated that metformin intake was not associated with a decreased incidence of bladder cancer (HR = 0.82, 95% CI = 0.61–1.09; *P* = .17; *I*^2^ = 85%) or an increased overall survival in bladder cancer patients (HR = 0.83, 95% CI = 0.47–1.44; *P* = .50; *I*^2^ = 64%).

**Conclusion:**

The present meta-analysis indicated that metformin intake could improve the prognosis of bladder cancer patients. Further prospective cohort studies and mechanistic studies are still required to determine the precise role of metformin in the initiation and progression of bladder cancer.

## Abstract 2

Supplemental Digital Content is available in the text

## 1 Introduction

Bladder cancer occurs frequently and is a common and aggressive malignancy of the urinary system, which is clinically characterized by its progression, recurrence, metastasis, and drug resistance.^[1]^ Although neoadjuvant chemotherapy, surgery, and postoperative chemotherapy constitute a comprehensive treatment regimen that can improve survival and quality of life, most patients experience recurrence and metastasis.^[2]^ Moreover, owing to the lifetime need for monitoring tumor recurrence, patients with bladder cancer usually have poor quality of life and the typical cost of the disease from diagnosis to death is one of the highest among all cancers.^[3]^

Diabetes mellitus (DM), a metabolic disease that affects patients worldwide, is associated with elevated cancer incidence and a worse prognosis.^[4–6]^ Fortunately, many researchers have found that patients with type 2 DM (T2DM) treated with metformin exhibited generally lower incidence of cancers and better long-term oncologic outcomes than those treated with other drugs.^[7–11]^ Metformin is a biguanide used as a first-line oral antidiabetic therapy for T2DM with an excellent safety profile. The drug can also be safely combined with other antidiabetic agents.^[12,13]^ However, there was no consensus as to whether the metformin can reduce the incidence or improve the oncological outcomes of bladder cancer.

Herein, we systematically performed a meta-analysis to explore the association of metformin intake with bladder cancer risk and oncologic outcomes in T2DM patients.

## 2 Methods

This study protocol was conducted according to the PRISMA (Preferred reporting items for systematic review and meta-analyses) statement^[14]^ (Supplementary Table 1) and approved by the institutional review board at Xiangya Hospital of Central South University before initiation. The need for ethical standard approval or informed consent was waived because of the nature of the research design.

### 2.1 Search strategy

In accordance with the PRISMA guidelines, a systematic review of literature was performed in December 2017 using PubMed (ncbi.nlm.nih.gov/pubmed), Embase (embase.com), and the Cochrane Central Search Library (cochraneli-brary.com). Search terms used included: [“Metformin” (Mesh) OR (metformin OR biguanides OR “Glucophage”)] AND [“Urinary Bladder Neoplasms” (Mesh) OR (bladder OR urinary bladder)] AND (neoplasms OR cancer OR carcinoma OR tumor). All abstracts and review articles on this topic were reviewed, and references of original studies were identified by manual search.

### 2.2 Inclusion/exclusion criteria

Eligible studies had to meet the following selection criteria: randomized controlled trials or cohort studies with a controlled group; studies evaluating the association between metformin use and bladder cancer; the report contained significant information about metformin use and bladder cancer susceptibility, recurrence-free survival (RFS), progression-free survival (PFS), cancer-specific survival (CSS), and overall survival (OS); sufficient information was provided to estimate relative risk (RR) or the hazard ratio (HR) with a 95% confidence interval (95% CI); studies published in English. Exclusion criteria were as follows: research in the form of case reports, reviews, case series, editorials, and letters; studies with insufficient data to estimate RR or HR with related 95% CI; (3) nonhuman researches.

### 2.3 Data extraction and quality assessment

Data of identified studies were extracted by 2 independent reviewers (J.H. and J.B.C.). Disagreement was resolved during a consensus with a third reviewer (X.Z.). The data from the literature and demographics were extracted individually. We extracted the following information: author, year of publication, country of study, study type, tumor stage, definition of exposure or intervention, sample size, age, survival analysis, adjusted variables, duration, follow-up, and data of survival analyses and references. An HR and its 95% CI were used to evaluate the association between metformin intake and incidence, RFS, PFS, CSS, and OS of bladder cancer. If available, the HRs with their 95% CIs and *P* values were collected from the original articles. If not available, HRs and their 95% CIs were calculated based on methods by Tierney et al.^[15]^ The quality of studies was evaluated using the Newcastle-Ottawa Scale (NOS) by 2 reviewers independently.^[16]^ Score of 7 to 9 was defined as a high-quality study, and a score <7 was defined as a low-quality study.

### 2.4 Statistical analysis

For time-to-event outcomes, we pooled the HRs with their 95% CIs to investigate the correlation between metformin intake and bladder cancer. We extracted the HRs with their 95% CIs directly when they were available in the article. Otherwise, we estimated them based on the related data or Kaplan-Meier survival curves according to the method described by Tierney et al.^[15]^ To avoid errors in calculation, 2 independent authors finished this process. Statistical heterogeneity among studies was checked using a formal *Q*-statistic as well as *I*-squared (*I*^2^). The degree of heterogeneity was measured by the value of *I*^2^ (*I*^2^ < 25%: no heterogeneity; *I*^2^ = 25%–50%: moderate heterogeneity; *I*^2^ >50%: large heterogeneity). When the heterogeneity was large, a random-effects model was used. Otherwise, the fixed-effects model was used. The level of statistical significance was set at 0.05.^[17]^ Although we could not evaluate publication bias by Begg funnel plot or Egger test^[18,19]^ because of the small number of included studies, we conducted a sensitivity analysis by the leave-one-out cross validation to assess the stability of the present meta-analysis results. The meta-analyses were performed using Review Manager (RevMan) software version 5.3 (The Nordic Cochrane Centre, The Cochrane Collaboration, Copenhagen).

## 3 Results

### 3.1 Study selection and characteristics

**Figure 1:**
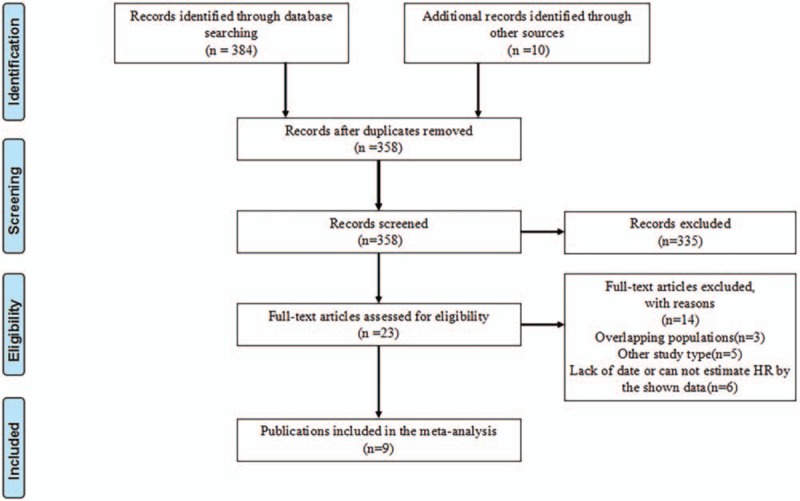
Flow chart of studies selection. HR = hazard ratio.

Finally, a total of 394 records were retrieved through databases. According to the inclusion and exclusion criteria, our present meta-analysis includes 9 studies,^[20–28]^ that comprehensively investigated the association between metformin intake, bladder cancer risk, and oncologic outcomes (Fig. 1). Specifically, data were available from 5 studies on metformin and bladder cancer susceptibility, 4 studies on metformin and RFS, 2 studies on metformin and PFS, 2 studies on metformin and CSS, and 3 studies on metformin and OS. The main characteristics of the eligible studies are summarized in Table 1. Results of our meta-analysis are summarized in Table 2. For the quality assessment, the NOS scores of the individual cohort studies ranged from 7 to 8. The details are listed in Supplementary Table 1.

**Table 1:**
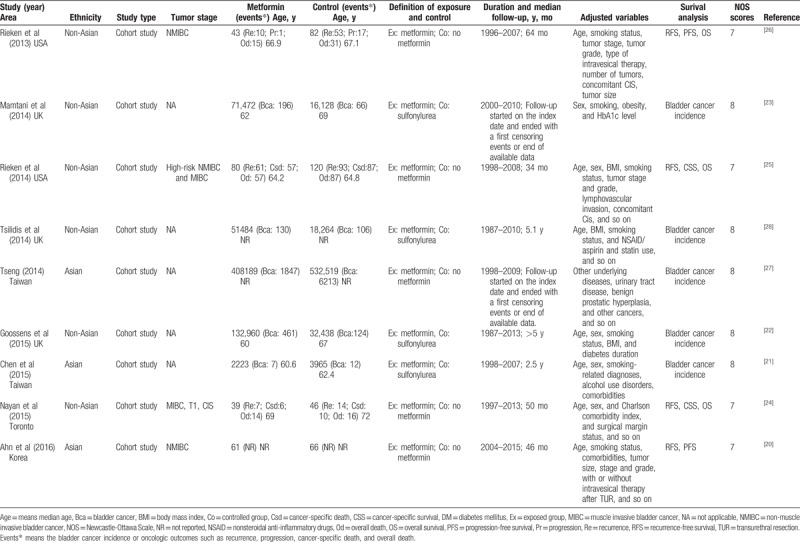
Characteristics of included retrospective studies.

**Table 2:**
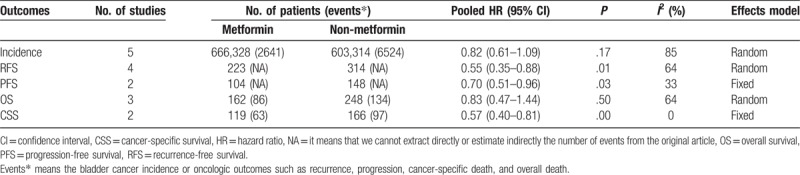
Analysis of metformin intake and bladder cancer oncologic outcomes.

### 3.2 Metformin intake and bladder cancer susceptibility

**Figure 2:**
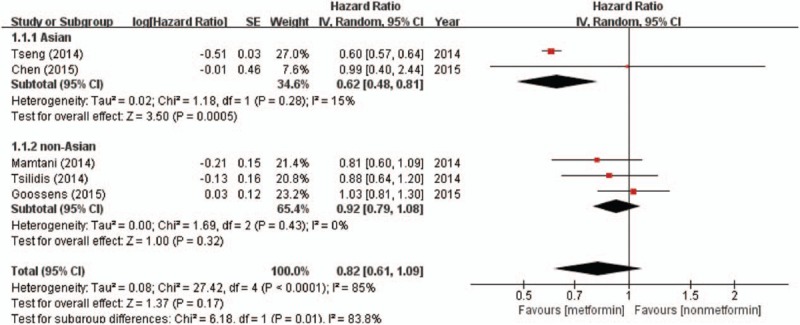
Forest plot of hazard ratio for bladder cancer incidence in patients with type 2 diabetes mellitus. CI = confidence interval, SE = standard error.

Five studies reported the association between metformin intake and bladder cancer susceptibility, which involved 1,269,642 patients (metformin intake 666,328 vs. non-metformin intake 603,314). There was significant heterogeneity between these studies (*I*^2^ = 85%; *P* < .01), so a random-effects model was used in the analysis. Heterogeneity may be caused by district and ethnicity differences, as well as the small number of included studies. Accordingly, a subgroup analysis was conducted based on ethnicity. Overall, metformin use was not associated with a decreased incidence of bladder cancer (HR = 0.82, 95% CI = 0.61–1.09; *P* = .17). A subgroup analysis by different ethnicities demonstrated that metformin intake had a significant association with bladder cancer among Asian patients (HR = 0.62; 95% CI = 0.48–0.81; *P* < .01). However, such an association was not observed for non-Asian patients (HR = 0.90, 95% CI = 0.79–1.08; *P* = .32) (Fig. 2).

### 3.3 Metformin intake and RFS and PFS of bladder cancer

**Figure 3:**
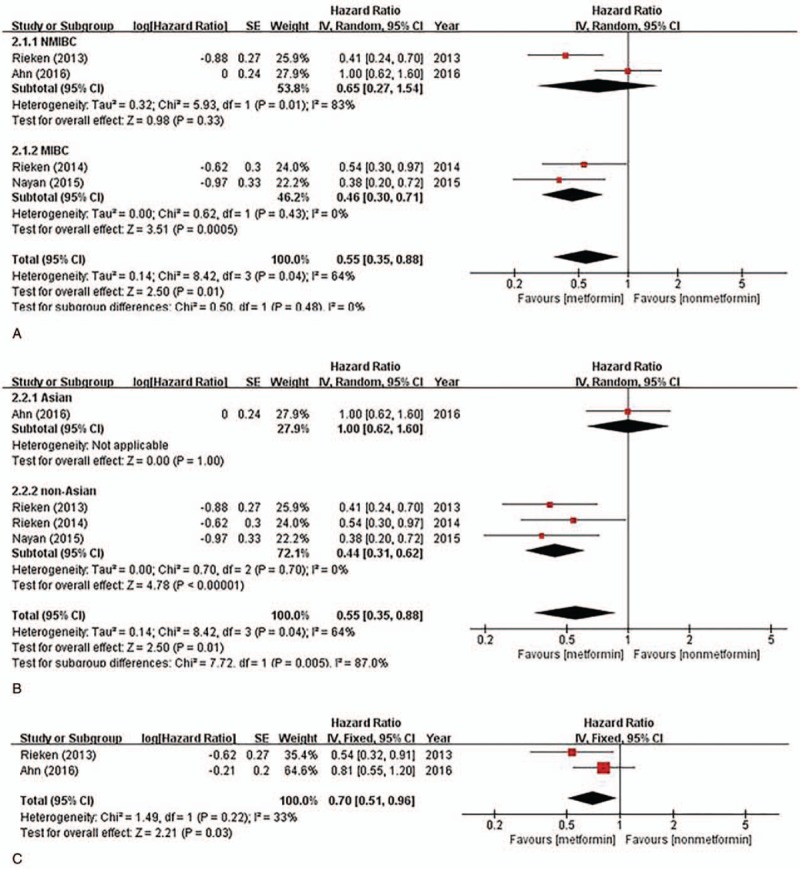
Forest plot of HR for recurrence-free and progress-free survival. (A) Association between metformin intake and recurrence-free survival sub-grouped by tumor stage; (B) association between metformin intake and recurrence-free survival sub-grouped by ethnicity; (C) association between metformin intake and progression-free survival. The diamond indicates the pooled HR value. CI = confidence interval, HR = hazard ratio, MIBC = muscle invasive bladder cancer, NMIBC = non-muscle invasive bladder cancer, SE = standard error.

Four studies reported the association between metformin intake and RFS of bladder cancer, which involved a total of 537 patients (metformin intake [223] vs. non-metformin intake [314]). There was significant heterogeneity between these studies (*I*^2^ = 64%; *P* = .04), so a random-effects model was used in the analysis. Heterogeneity may be attributed to different tumor stages, follow-up time, district, and ethnicity. Thus, a subgroup analysis was conducted according to these factors. Overall, metformin intake was associated with an increased RFS of bladder cancer (HR = 0.55, 95% CI = 0.35–0.88; *P* = .01). Subgroup analysis by different tumor stage demonstrated that metformin use had an increased RFS for muscle invasive bladder cancer (MIBC) (HR = 0.46; 95% CI = 0.30–0.71; *P* *<* .01), whereas such an association was not observed for non-muscle invasive bladder cancer (NMIBC) (HR = 0.65, 95% CI = 0.27–1.54; *P* = .33) (Fig. 3A). A subgroup analysis of the different ethnicities demonstrated that metformin use had an increased RFS for non-Asians (HR = 0.44; 95% CI = 0.31–0.62; *P* < .01), whereas such an association was not observed for Asians (HR = 1.00, 95% CI = 0.62–1.60; *P* = 1.00) (Fig. 3B). Two studies reported the association between metformin intake and PFS of bladder cancer. There was no statistical heterogeneity between the trials (*I*^2^ = 33%; *P* = .22), so a fixed-effects model was used in the analysis. Overall, metformin intake was associated with an increased PFS of bladder cancer (HR = 0.70, 95% CI = 0.51–0.96; *P* = .03) (Fig. 3C).

### 3.4 Metformin intake and OS and CSS of bladder cancer

**Figure 4:**
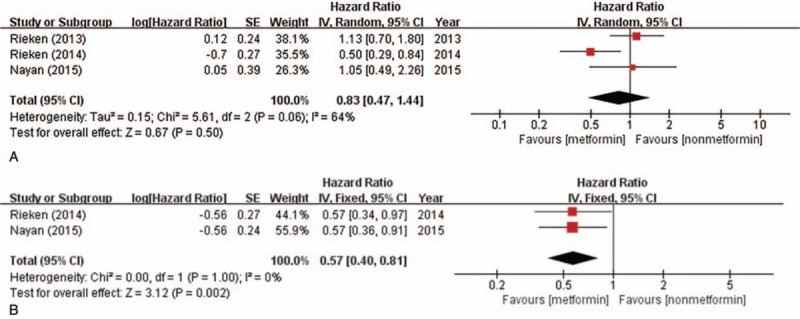
Forest plot of HR for overall survival and cancer-specific survival. (A) Association between metformin intake and overall survival; (B) Association between metformin intake and cancer-specific survival. The diamond indicates the pooled HR value. CI = confidence interval, SE = standard error.

Three studies reported the association between metformin intake and OS of bladder cancer. There was statistical heterogeneity between the trials (*I*^2^ = 64%; *P* = .06), so a random-effects model was used in the analysis. Overall, no significant association was observed between metformin use and OS of bladder cancer (HR = 0.83, 95% CI = 0.47–1.44; *P* = .50) (Fig. 4A). Two studies reported the association between metformin intake and CSS of bladder cancer. There was no statistical heterogeneity between the trials (*I*^2^ = 0%; *P* = 1.00), so a fixed-effects model was used in the analysis. Overall, metformin intake was associated with an increased CSS of bladder cancer (HR = 0.57, 95% CI = 0.40–0.81; *P* = .002) (Fig. 4B).

### 3.5 Sensitivity analysis of cohort studies

**Figure 5:**
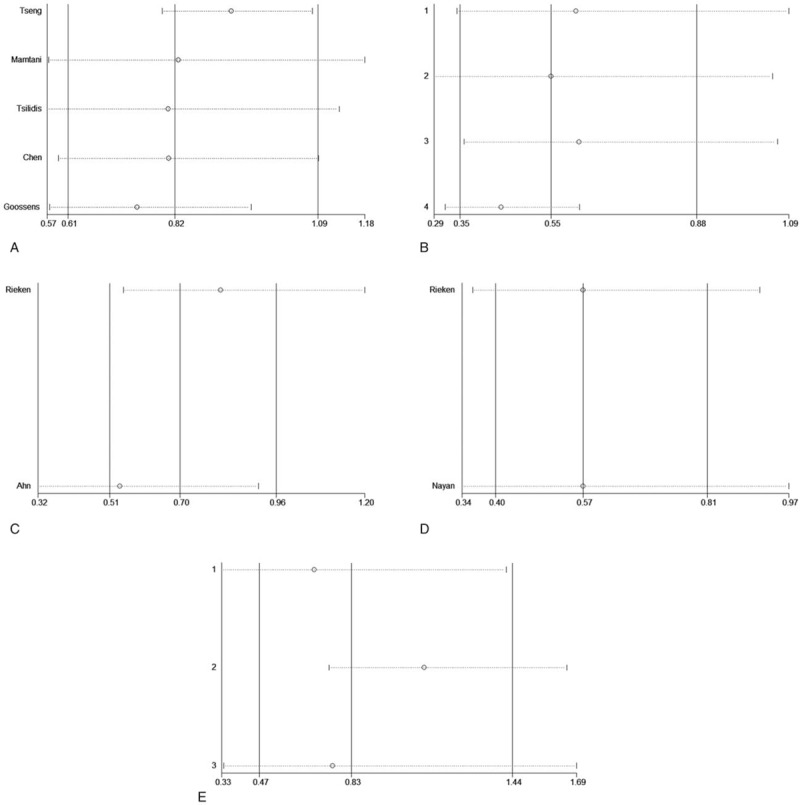
Sensitivity analysis for (A) the incidence, (B) recurrence-free survival, (C) progression-free survival, (D) cancer-specific survival, and (E) overall survival.

A sensitivity analysis was performed by leave-one-out cross validation to assess the stability of present meta-analysis results. After removing these studies associated with heterogeneity, the overall HR did not change significantly, so the meta-analysis was fairly stable and convincing (Fig. 5A–E).

## 4 Discussion

Metformin can play a major role in suppressing cancer through the following mechanisms. First, it activates AMP-activated protein kinase (AMPK) to lower the blood glucose level, which can indirectly inhibit the growth of tumor cells.^[29]^ Meanwhile the activation of AMPK also downregulates the mammalian target of rapamycin (mTOR), which eventually induces growth inhibition of cancer cells and protein synthesis arrest.^[30]^ A study by Zhang et al^[31]^ showed that metformin inhibits the growth of bladder cancer cells via indirect activation of AMPK, which in turn suppresses the mTOR/p70 S6 kinase-1 (S6K1) pathway in 253J and RT4 bladder cancer cell lines. Second, it can reduce the plasma level of insulin and downregulate Insulin Receptor Substrate-1 (IRS-1), which results in inactivation of downstream insulin-associated signaling pathways like PI3K-AKT/Protein Kinase B (PKB) and Ras-MAPK to inhibit tumor growth.^[32,33]^ In line with this mechanism, Wang et al^[34]^ demonstrated that human bladder cancer cells transfected with hsa-miR-96 inhibitor significantly reduced the growth of bladder cancer cells through reduction of mRNA and protein levels of IRS-1. Third, metformin can promote cell apoptosis through both caspase-dependent and caspase-independent mechanisms.^[35,36]^ More importantly, it can decrease suvivin, which is a potentially significant protein with a crucial role in treatment and prognosis of bladder cancer.^[37]^ Fourth, metformin can enhance the action of chemotherapeutic drugs such as cisplatin and doxorubicin.^[38,39]^ Other possible antitumor functions of metformin involve autophagy mechanisms and immune mechanisms.^[40–42]^

Our present meta-analysis, that included 9 retrospective cohort studies, comprehensively investigated the effect of metformin intake on incidence and oncologic outcomes of bladder cancer. There is a discrepancy among the results of these studies. Studies by Tsilidis et al,^[28]^ Mamtani et al,^[23]^ Goossens et al,^[22]^ and Chen et al^[21]^ demonstrated that metformin use was not associated with a decreased incidence of bladder cancer. In contrast, data from the study by Tseng et al^[27]^ showed that metformin had a protective effect on the incidence of bladder cancer. The current meta-analysis, which pooled results from these 5 studies, revealed no significant association between metformin intake and bladder cancer susceptibility. This negative result can be explained in a number of ways. First, there was a clear regional difference in the incidence of bladder cancer,^[43]^ which was caused by different risk factors of bladder cancer, such as smoking and industrial chemicals.^[44]^ Similarly, a subgroup analysis found that metformin use had a protective effect on the incidence of bladder cancer in Asians. However, this protective effect on morbidity was not observed for non-Asians. Meanwhile, we found that heterogeneity decreased significantly. (Asian group: *I*^2^ = 15%; non-Asian group: *I*^2^ = 0%). Therefore, we thought that the difference in ethnicity caused this large heterogeneity in incidence. Second, different databases had different coding and registration practices for patients with bladder cancer, which may have resulted in relatively high and stable incidence rates and a low mortality to incidence ratio.^[45]^ Third, differences among the clinical characteristics of patients between exposed and nonexposed groups, such as age, body mass index (BMI), and glycemic control, may have affected the incidence and progression of bladder cancer.^[46,47]^ After we checked the heterogeneity by the leave-one-out cross validation, we found no heterogeneity existed when we removed the study performed by Tseng.^[27]^ We found that many baseline characteristics differed between these 2 groups. The metformin group was characterized by a smaller proportion of patients aged ≥70 years, a higher proportion of males, and a lower frequency of comorbidities. These differences in baseline characteristics could influence the incidence of bladder cancer and lead to false-positive results for the nonexposed group, which could overestimate the anticancer effect of metformin. Fourth, the antitumor effect of metformin was dose-dependent and treatment time-dependent, which was shown by Tseng.^[27]^ However, studies included in our meta-analysis did not specify the treatment time and dose of metformin. Hence, more comprehensive and larger multicenter clinical studies are imperative to confirm whether metformin can reduce the risk of bladder cancer.

According to the European Organization for Research and Treatment of Cancer (EORTC) database, the 5-year recurrence of NMIBC ranged from 31% to 78%.^[1]^ Therefore, bladder cancer is a disease with a high recurrence and progression rate. Recently, there was no consensus as to whether the metformin can improve the oncological outcomes of bladder cancer. Studies by Nayan et al^[24]^ and Rieken et al^[25]^ indicated that metformin use could prolong RFS and PFS in patients with bladder cancer; however, such protective effects were not reported in a study by Ahn et al.^[20]^ The present meta-analysis suggested that metformin intake could improve RFS and PFS of bladder cancer. In view of the significant heterogeneity of RFS, we conducted a subgroup analysis based on different tumor stages and ethnicity. There was no heterogeneity after conducting subgroup analysis by ethnicity, which means that different ethnicity was a main reason for this heterogeneity. The results showed that metformin could extend bladder cancer RFS for MIBC patients and non-Asians. However, this protective effect was not observed for NMIBC patients. The follow-up time of studies in our meta-analysis ranged from 34 to 64 months. Therefore, at the end of follow-up, some of the bladder cancer end-stage events, such as recurrence, progression, and death, could not been observed or recorded for NMIBC patients in our included studies. The short follow-up time might underestimate metformin's protective effect on RFS and PFS of patients with bladder cancer. Accordingly, we found that no heterogeneity existed when we removed the study performed by Ahn et al.^[20]^ Their research indicated that poor baseline and post-operative glycemic control were poor prognosis predictors and indicated that metformin intake had no impact on disease recurrence and progression in bladder cancer patients. This difference might be caused by differences in ethnicity. Additionally, 2 studies reported on CSS and both demonstrated that metformin intake could improve the CSS of bladder cancer. Consistent with these individual studies, the pooled results of our meta-analysis also suggested that metformin intake could improve the CSS of bladder cancer. However, our meta-analysis did not uncover an association between metformin use and improved OS. Differences in risk of noncancerous mortality between metformin users and nonusers as well as a short-term follow-up time may confound this association.

There are several limitations that should be noted in our current meta-analysis. First, the included data were retrieved from retrospective cohort studies. This may have potentially led to some selection bias. Second, publication quantity was limited for several oncologic outcome analyses, which restrained our ability to conduct a subgroup analysis. Third, all eligible studies were restricted to English, and the exclusion of other languages studies may have increased publication bias. Fourth, several potential confounding factors were not considered, such as age, sex, smoking habits, drinking status, and environmental factors. Moreover, several HRs are calculated based on the data extracted from the survival curve, which may also induce some errors.

## 5 Conclusion

This meta-analysis supported a favorable anticancer role of metformin in survival outcomes associated with bladder cancer. Although metformin was not found to decrease the incidence of bladder cancer, our current results demonstrated that metformin intake improved the patient's RFS, PFS, and CSS. Further clinical and mechanistic studies are still required to determine the precise role of metformin in the initiation and progression of bladder cancer.

## Author contributions

J.H., J.B.C., and X.Z. have made substantial contributions to design the study. X.B.Z. and L.L. have screened papers and conducted the quality rating and meta-analysis. J.H., J.B.C., and Y.C. searched and selected the trials and extracted data. J.H., J.C., Y.W.Z., and W.B.R. performed the analysis. J.H. and J.B.C. wrote the manuscript. X.B.Z. and H.C. reviewed the manuscript. J.H., J.C., and X.B.Z. proofread the final version. All authors read and approved the final article.

**Conceptualization:** He-qun Chen.

**Data curation:** Yu Cui, Xu Zhou, Long-fei Liu.

**Formal analysis:** Ye-wen Zhu, Wen-biao Ren.

**Writing – original draft:** Jiao Hu, Jin-bo Chen.

**Writing – review & editing:** Jiao Hu, Jin-bo Chen, Xiong-bing Zu.

## Supplementary Material

Supplemental Digital Content
