# Supplementary material for: Association of metformin intake with bladder cancer risk and oncologic outcomes in type 2 diabetes mellitus patients: A systematic review and meta-analysis
Source: Medicine (Baltimore). 2018 Jul 27;97(30):e11596. doi: 10.1097/MD.0000000000011596 (PMC6078654; doi:10.1097/MD.0000000000011596)
Supplement: Supplemental Digital Content [file medi-97-e11596-s001.doc]

**Supplementary Table 2**. The Newcastle-Ottawa Scale (NOS) for assessing the quality of cohort studies.

| **Study (year)** | **Seletion** | | | | **Comparability** | | **Assessment of outcome** | | | **Total quality score** |
| --- | --- | --- | --- | --- | --- | --- | --- | --- | --- | --- |
|  | **Representative-ness of the exposed cohort** | **Ascertainment of exposure** | **Selection of Controls** | **Demonstration that outcome of interest was not present at start of study** | **study controls for the most important factors** | **study controls for any additional factor** | **Assessment of outcome** | **Was follow-up long enough for outcomes to occur** | **Adequacy of follow up of cohort** |  |
| Rieken (2013) | * | * | * | / | * | * | * | * | / | 7 |
| Mamtani (2014) | * | * | * | / | * | * | * | * | * | 8 |
| Rieken (2014) | * | * | * | / | * | * | * | * | / | 7 |
| Tsilidi (2014) | * | * | * | / | * | * | * | * | * | 8 |
| Tseng (2014) | * | * | * | / | * | * | * | * | * | 8 |
| Goossens (2015) | * | * | * | / | * | * | * | * | * | 8 |
| Chen (2015) | * | * | * | / | * | * | * | * | * | 8 |
| Nayan (2015) | * | * | * | / | * | * | * | * | / | 7 |
| Ahn (2016) | * | * | * | / | * | * | * | * | / | 7 |
